# Quasi-specific access of the potassium channel inactivation gate

**DOI:** 10.1038/ncomms5050

**Published:** 2014-06-09

**Authors:** Gaurav Venkataraman, Deepa Srikumar, Miguel Holmgren

**Affiliations:** 1 Molecular Neurophysiology Section, National Institute of Neurological Disorders and Stroke, National Institutes of Health, Bethesda, Maryland 20892, USA; 2 Present address: CNC Building, Stanford University, Palo Alto, California 94304, USA

## Abstract

Many voltage-gated potassium channels open in response to membrane depolarization and then inactivate within milliseconds. Neurons use these channels to tune their excitability. In Shaker K^+^ channels, inactivation is caused by the cytoplasmic amino terminus, termed the inactivation gate. Despite having four such gates, inactivation is caused by the movement of a single gate into a position that occludes ion permeation. The pathway that this single inactivation gate takes into its inactivating position remains unknown. Here we show that a single gate threads through the intracellular entryway of its own subunit, but the tip of the gate has sufficient freedom to interact with all four subunits deep in the pore, and does so with equal probability. This pathway demonstrates that flexibility afforded by the inactivation peptide segment at the tip of the N-terminus is used to mediate function.

## Abstract (web summary)

Inactivation of Shaker potassium channels is caused by one of the four cytoplasmic amino termini, termed the inactivation gate. Here, Venkataraman *et al*. show that a single gate threads through the intracellular entryway of its own subunit and interacts with all four subunits deeper in the pore, demonstrating the function of this N-terminus.

## Graphical abstract

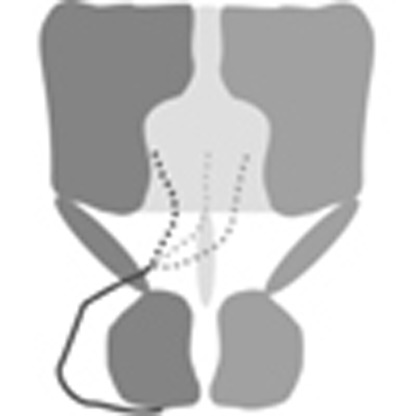

Voltage-activated potassium (K_V_) channels are potassium selective integral membrane proteins formed by the assembly of four homologous subunits^1^,^2^. In response to a membrane depolarization, K_V_ channels open, allowing K^+^ to permeate. In many members of K_V_ channels, sustained depolarization leads to fast inactivation caused by an N-terminus gate^3^,^4^. Excitable cells utilize inactivating K_V_ channels to shape their action potentials and adjust their firing patterns^5^,^6^,^7^,^8^,^9^,^10^. It has been demonstrated that the inactivation gate is fully extended during inactivation such that its hydrophobic N-terminus tip interacts with residues deep in the intracellular cavity^11^,^12^, blocking the permeation of K^+^^13^. Away from the tip, hydrophilic regions of the N-terminus interact with the cytosolic protein surface at the intracellular entryway^14^,^15^,^16^.

By the nature of their tetrameric architecture, inactivating K_V_ channels have four inactivation gates, four sets of interacting residues deep in the cavity and four intracellular entryways. Yet, N-type inactivation is produced by the binding of only one inactivation gate in the pore^17^,^18^.

Here we ask: is the pathway of a single inactivation gate into its inactivating position specific to the subunit to which it belongs? We show that the inactivation gate always threads through the intracellular entryway above its own T1 domain, but interacts with all the four subunits at its site of action deep in the pore.

## Results

### Construction of a Shaker K_V_ concatemer channel

**Figure 1:**
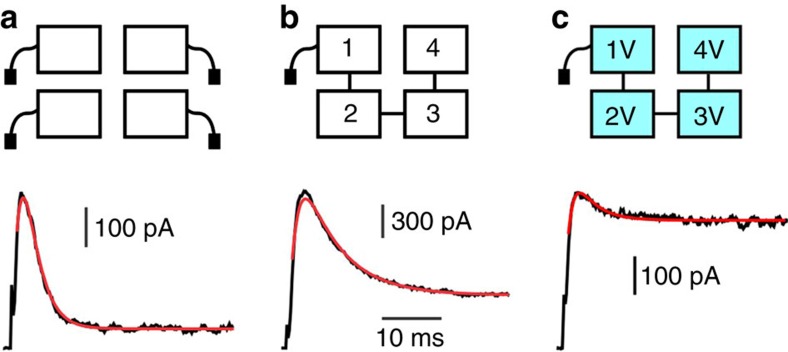
Effects of concatenation and I470V mutations on inactivation. (**a**) Shaker homotetramer. Cartoon represents a Shaker homotetramer channel having four inactivation gates (top) with a representative K^+^ current in response to a voltage step to +60 mV from a holding potential of −80 mV displayed below. Red line overlaying the trace represents a fit of the inactivation process to a Markov kinetic model parameterized by the time constants *k*_on_ and *k*_off_. Average values were: *k*_on_=430±70 s^−1^; *k*_off_=21±2 s^−1^. Horizontal bar for all panels: 10 ms; Vertical bar: 100 pA. (*n*=5 patches, 43 traces). (**b**) Shaker concatemer having one inactivation gate. Cartoon (top) with a representative K^+^ current shown below. Best fit parameter values were: *k*_on_=108±21 s^−1^ and *k*_off_=40±9 s^−1^. We noticed that *k*_off_ of wild-type concatemer was ~ × 2 faster than Shaker homotetramer. Vertical bar: 300 pA. (*n*=7 patches, 68 traces). (**c**) Shaker concatemer having one inactivation gate and four subunits with I470V mutation. Cartoon (top) with a representative K^+^ current shown below. Best fit parameter values were: *k*_on_=90±3 s^−1^; *k*_off_=163±66 s^−1^. Vertical bar: 100 pA. (*n*=10 patches, 114 traces).

To study the pathway of a single inactivation gate, we constructed a Shaker concatemer channel having only one free N-terminus. Figure 1b shows a current trace from concatemer channels in response to a depolarization step. Figure 1a shows a current trace from Shaker homotetramers in response to the same depolarization step. Solid red lines overlaying the traces represent fits of a Markov kinetic model to the current traces. As expected, the extent of inactivation in the concatemers was less than that of the wild-type channels, and the relaxation slower. Quantitatively, we observed a fourfold reduction in the on rate of inactivation (*k*_on_) for concatemer channels relative to Shaker homotetramers, consistent with the presence of only one inactivation gate relative to four.

### Site of inactivation gate action in the pore

We begin by asking: does the site of action in the pore depend on the subunit to which the inactivation gate belongs? In K_V_ homotetramers, it has been previously established that inactivation gate’s site of action is at the intracellular cavity of the channel^11^,^12^. In particular, mutating Shaker’s position 470 from isoleucine to valine (I470V) dramatically reduced the extent of N-type inactivation^11^,^19^. Mutating I470V in all the four subunits of Shaker concatemer (Fig. 1c) reproduced the effect on inactivation previously reported in I470V Shaker homotetramer.

**Figure 2:**
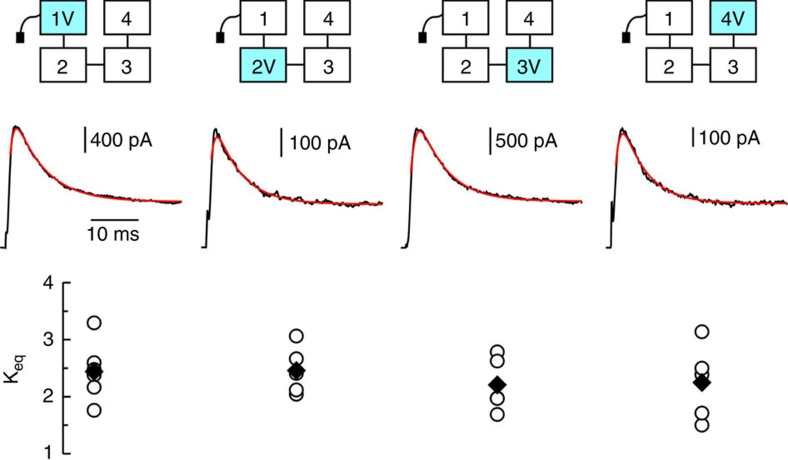
A single subunit mutated to I470V is not sufficient to reproduce the reduction in inactivation seen in the concatemer having four I470V subunits. Cartoons of characterized mutants are shown at top, vertically aligned with their corresponding traces and K_eq_ values. Cyan colour represents a subunit mutated to I470V. Middle: representative current traces in response to a voltage step to +60 mV from a holding potential of −80 mV, overlaid with best-fits to a Markov kinetic model. Bottom: K_eq_ values. Horizontal bar: 10 ms; Vertical bars (from left to right): 400, 100, 500 and 100 pA. Open circles represent individual experiments and filled diamonds correspond to their averages.

We reasoned that if the inactivation gate interacts with only a single and specific subunit inside the pore to produce inactivation, then a single I470V mutation at this interacting subunit would produce a reduction in inactivation comparable with that observed when all the four subunits are mutated to I470V. Current traces of Shaker concatemers with a single subunit mutated to I470V are shown in Fig. 2. No individual I470V mutation reproduced the dramatic effect observed when all the four subunits were mutated to I470V. Rather, none of the individual I470V subunit mutations produced significant reduction in the extent of inactivation as compared with the nonmutated concatemer. These results demonstrate that despite belonging to a particular subunit, a single inactivation gate does not have an exclusive site of action in the cavity. In fact, the inactivating particle samples all possible sites of action with roughly equal probability.

**Figure 3:**
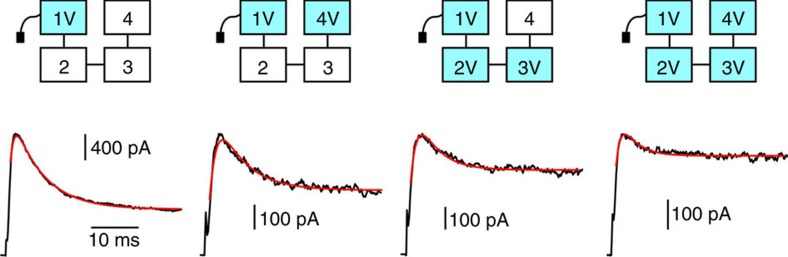
Multiple I470V mutations are required to modify the energetics of N-type inactivation. Top: cartoons of selected characterized mutants, where cyan colour represents a subunit mutated to I470V. Bottom: representative current traces from mutants cartooned above. All currents are in response to a voltage step to +60 mV from a holding potential of −80 mV, and overlaid with best-fits to a Markov kinetic model. Horizontal bar: 10 ms; Vertical bars (from left to right): 400, 100, 100 and 100 pA.

**Figure 4:**
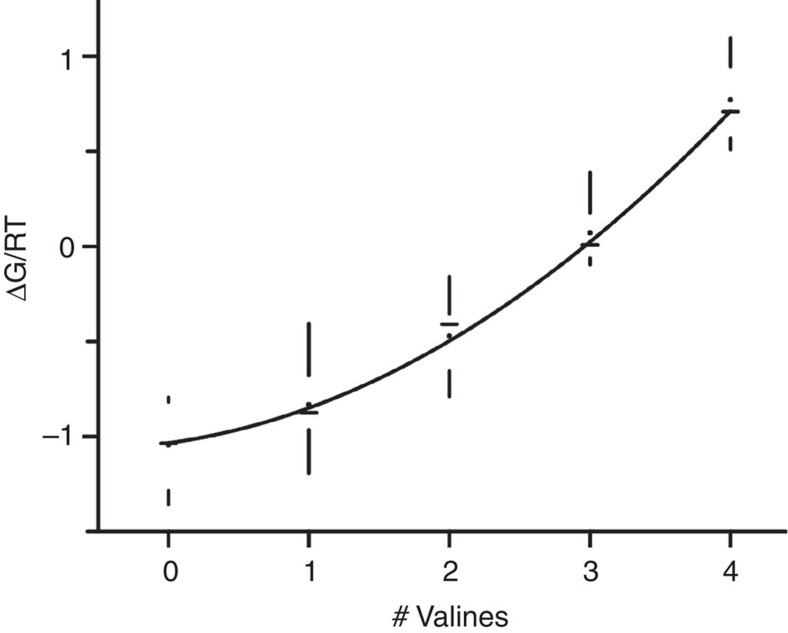
Inactivation free energy as a function of the number of subunits mutated to I470V. Free energy was calculated from *k*_on_ and *k*_off_, obtained from the Markov kinetic model and plotted in RT units. The following mutants are represented in the plot: wild-type concatemer; Single I470V mutations: 1V; 2V; 3V; 4V. Two I470V mutations: 1V4V; 2V4V. Three I470V mutations: 1V2V3V; 2V3V4V. All subunits with I470V mutations: 1V2V3V4V. The solid line represents a fit to a model in which the single inactivation gate can bind to one of four possible binding sites at the cavity. If there is a valine present, the apparent affinity of the remaining isoleucines is increased by 8% (see Methods). Data are shown in box plot format (horizontal lines represent the medians, dots correspond to averages and the range of the samples is denoted by vertical lines). *n*_(0V)_=7 patches, *n*_(1V)_=22 patches, *n*_(3V)_=11 patches and *n*_(4V)_=10 patches.

Given that mutating a single subunit to I470V causes no change in inactivation, we ask: what is the effect of mutating two or three subunits to I470V? Figure 3 shows representative traces from concatemer channels having one, two, three or four subunits mutated to I470V. A stepwise increase in the number of valines in the cavity produced a corresponding decrease in the extent of inactivation. If each site of action acts independently on the single inactivation gate, we would expect the free energy of inactivation (ΔG) to change linearly as a function of the number of subunits mutated to I470V. Figure 4 shows that this relationship is not linear; the physical principles governing this nonlinearity are unknown. The solid line represents a model in which the presence of each additional I470V subunit increases the apparent affinity of the remaining wild-type I470 by ~8%.

### Access of the inactivation gate to the pore

**Figure 5:**
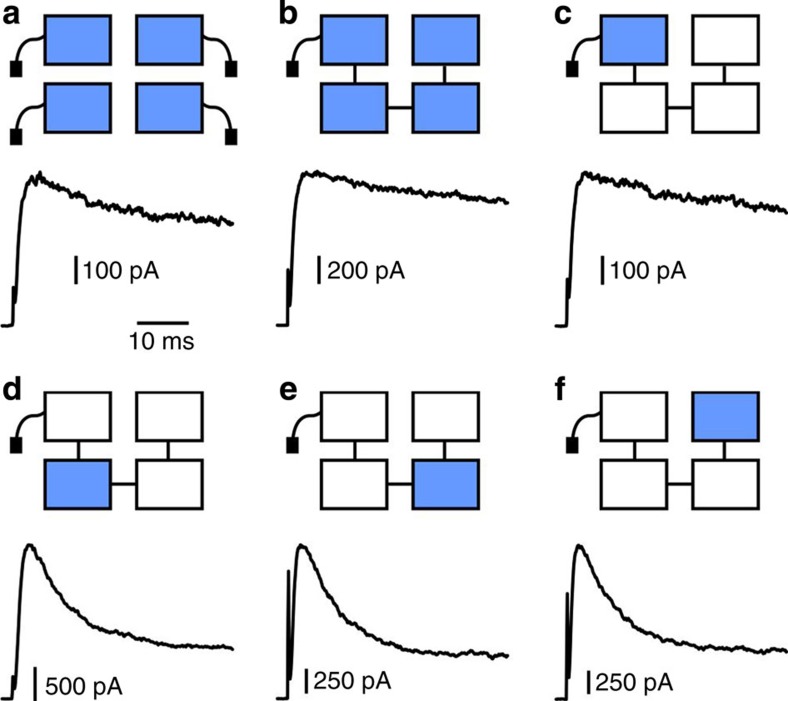
The inactivation gate selectively enters the intracellular vestibule. (**a**) Shaker homotetramer E192K D193K mutant. Cartoon (top) and representative current trace (below) in response to a voltage step to +60 mV from a holding potential of −80 mV. Horizontal bar for all panels: 10 ms. Vertical bar: 100 pA. (**b**) Shaker concatemer having all subunits mutated to E192K 193K. Cartoon (top) and representative current trace (below). Vertical bar: 200 pA. (**c**) Shaker concatemer having the E192K D193K mutation at only the free N-terminus containing subunit. Cartoon (top) and representative current trace (below). Vertical bar: 100 pA. (**d**–**f**), cartoons (top) and representative current traces (below) in response to a voltage step to +60 mV from a holding potential of −80 mV and their corresponding for: Shaker concatemer having the E192K D193K mutation at only subunit 2 (**d**), Shaker concatemer having the E192K D193K mutation at only subunit 3 (**e**) and Shaker concatemer having the E192K D193K mutation at only subunit 4 (**f**). Vertical bars (from left to right): 500, 250 and 250 pA. *n*=3 for all constructs.

Having shown that a single inactivation gate has sufficient freedom to interact with all subunits deep inside of the pore, we next ask: does the gate have sufficient freedom to enter the pore through multiple intracellular entryways? It has been shown previously that the N-terminus interacts electrostatically with residues in the T1 domain, on the outer part of the intracellular entryway windows^14^,^20^. We made charged mutations at two such window residues (E192K, D193K) in Shaker K_V_ homotetramer, and observed a dramatic effect on the extent and kinetics of inactivation (Fig. 5a). We observed that a concatamer having all subunits mutated to (E192K D193K) reproduced the effect seen in Shaker homotetramer exactly (Fig. 5b).

We next made single E192 D193K mutations at each subunit of our concatemer. We reasoned that if the N-terminus threads through a unique intracellular entryway, a single E192K D193K at this entryway’s subunit would reproduce the effect seen when E192K D193K is introduced into Shaker homotetramer. Strikingly, concatemers having a single E192K D193K mutation at the subunit containing the free N-terminus precisely reproduced the effect observed in Shaker E192K, D193K homotetramers (Fig. 5c). E192K D193K mutations at any of the three remaining subunits produced no effect on inactivation (Fig. 5d–f). These results demonstrate that the inactivation gate threads through the intracellular entryway above its own T1 domain.

## Discussion

**Figure 6:**
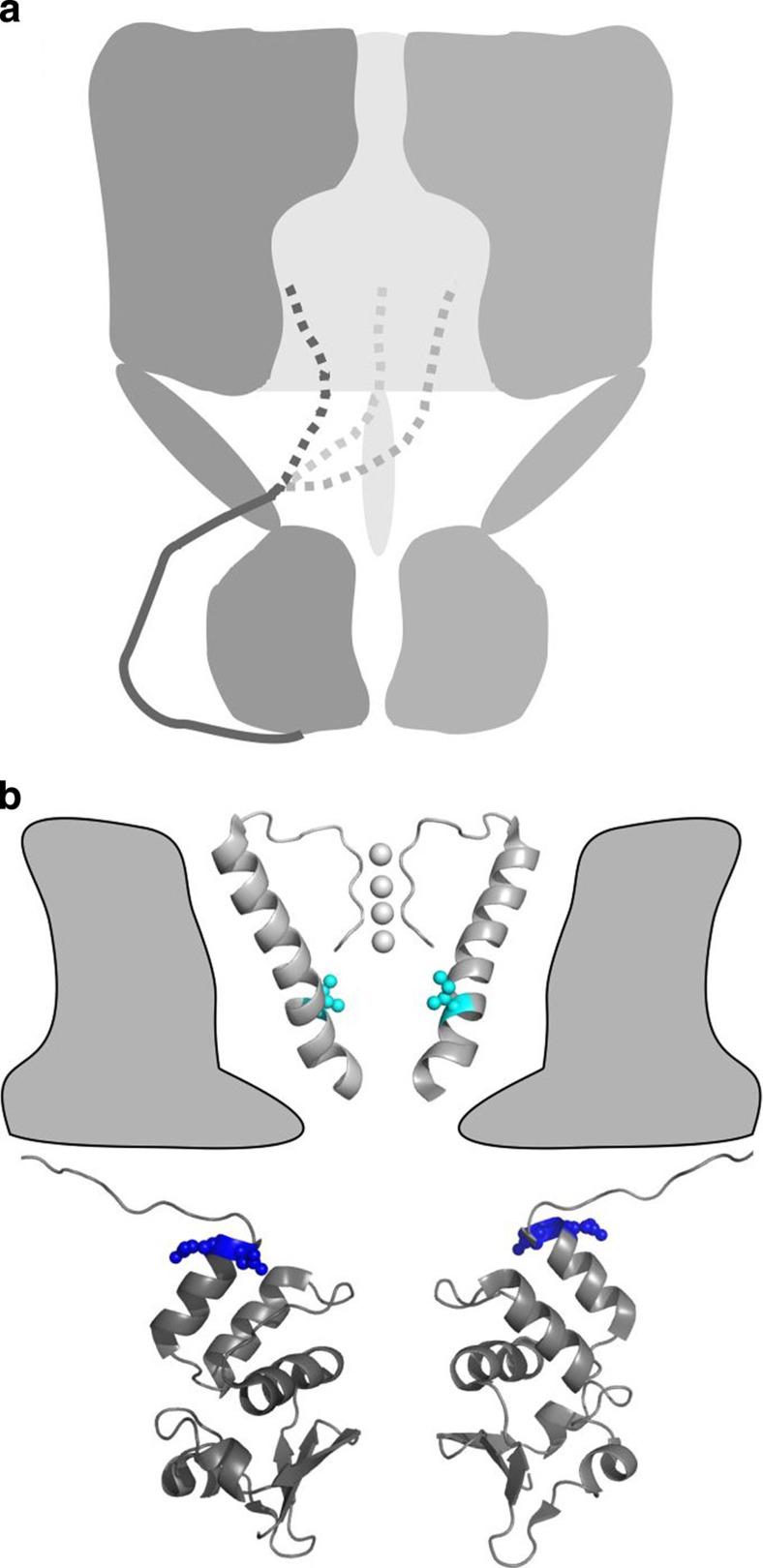
Access of K_V_’s inactivation gate. (**a**) Cartoon of the proposed inactivation gating mechanism. (**b**) Model of a K_V_ channel based on the ‘paddle-chimaera channel’ structure^30^. For clarity, the membrane region of the channel contains only two opposite selectivity filters and S6 transmembrane segments with their equivalent positions I470 in cyan. Similarly, two opposite T1 domains and their linkers are shown at the cytoplasmic region of the channel with equivalent positions E192 and D193 shown in blue.

It is well established that the Shaker inactivation gate is contained within the N-terminus of the channel^3^,^4^,^11^,^14^,^21^. Although K_V_ channels are tetramers^1^, inactivation is produced by a single N-terminus entering the intracellular cavity of the channel and blocking ion permeation following the opening of the voltage-dependent intracellular gate^13^,^17^,^18^,^22^. We have shown that a single inactivation gate always threads through the intracellular entryway above its own subunit, but may interact with any of the four subunits at its site of action deep in the pore (Fig. 6a). Our data do not distinguish the location of the inactivation gate when the channel is closed, nor its position as the channel transitions from its closed to open state.

We conclude that the inactivation gate relies crucially on both flexibility deep in the pore and spatially specific interactions at the T1 domain to produce inactivation (Fig. 6b). Near the intracellular entryways, the inactivation gate may have a secondary structure^23^,^24^ with multiple polar charges causing the pathway of the gate to be spatially specific^14^. During the ~50 ms that the inactivation gate resides in the intracellular cavity (*k*_off_ ~20 s^−1^ for WT channels; Fig. 1), the gate is likely to be in an ordered state stabilized by specific hydrophobic interactions between the tip of the N-terminus and the wall of the cavity^11^,^12^,^14^. Our data on the energetics of binding at the cavity suggest that the flexibility afforded by an unbound peptide segment at the tip of the N-terminus^20^,^24^,^25^ is used to sample all the four subunits deep in the pore and thereby mediate physiological effects. In mammalian K_V_1.1 channels, the position equivalent to Shaker 470 is targeted by RNA editing, resulting in an isoleucine to valine conversion^19^,^26^. This editing event varies largely within the nervous system^26^. Our data suggest that a change in excitability only occurs in neurons with a large percentage of K_V_1.1 mRNA edited, such that channels with three or four valines in the cavity are most abundant.

## Methods

### Shaker concatemer channels

All subunits were initially created in their own shuttle constructs, where unique restriction sites for concatemer construction were inserted. Subunits 1 and 2 were linked by AvrII (cctagg; introducing Pro and Arg); Subunits 2 and 3 were linked by SgrA1 (cgccggcga; introducing three Args); Subunits 3 and 4 were linked by KpnI (ggtacc; introducing Gly and Thr). All sequences of primers used in this study are shown in Table 1. All subunits contain the following background mutations: C301S, C308S and T449V^15^. The two cysteine mutations were originally removed to have a suitable cysteine-less channel to be used for cysteine modification experiments. These mutations have no functional consequences. T449V is a mutation that substantially reduces the extent of C-type inactivation^15^,^27^,which greatly simplifies our analysis of N-type inactivation. Subunit 1 contains the intact wild-type N-terminus conferring the inactivation gate to the concatemer. Subunits 2, 3 and 4 are the inactivation removed versions of Shaker (Δ6–46)^3^,^4^, which completely abolish fast inactivation.

### Mutagenesis

All mutations were performed using standard PCR techniques and subcloned into the GW1-CMV expression vector (Table 1). Individual subunit mutations were first subcloned into the shuttle construct, confirmed by sequencing and then inserted into the concatemer. All concatemers were subjected to a vast set of restriction digestion tests to verify that all subunits were in place and in order. Finally, all concatemer mutants were also sequenced to verify the proper number of containing mutations.

### DNA expression

All channel constructs’ DNA were expressed in HEK293 cells. DNA was transfected using a Nucleofector II (Amaxa Biosystem) system following their recommended protocol. Experiments were performed between 1–2 days after transfection.

### Experimental solutions and electrophysiological recordings

The intracellular solution was composed of (mM): 160 KCl, 0.5 MgCl_2_, 1 EGTA and 10 HEPES (pH=7.4). The extracellular solution contained (mM): 150 NaCl, 10 KCl, 1 MgCl_2_, 3 CaCl_2_, 10 HEPES (pH 7.4). All chemicals were purchased from SIGMA. Current recordings were obtained from inside-out excised patches^28^, using an Axopatch 200B amplifier (Axon Instruments). Currents were sampled at 10 kHz and filtered at 2 kHz. Protocol control and sampling were done using Clampex software and a Digidata 1200 AD/DA converter (Axon Instruments). Borosilicate glass (Harvard Instrument) pipettes were pulled to about 1.5–2.5 MΩ resistance (Sutter Instrument).

### Modelling ionic currents

Markov models were constructed with Matlab according to the following transition scheme: C_1_←C_2_←O←I. Simulated current was computed via I=iN*P_(O)_, where P_(O)_ is the probability of the channel being in the open state and iN is a single scale factor representing the number of channels in the patch multiplied by the unitary conductance for one channel. Traces were fit with the Levenberg–Marquardt algorithm via the Matlab program lsqnonlin. Transition rates leading to the open state were held fixed when fitting mutant channels’ current, after being estimated from ionic currents elicited by Shaker homotetramers and Shaker concatemers. Sensitivity analysis^29^ confirmed that these transition rates were not sensitive to changes in the extent of inactivation. Fits to mutant channels’ current had *k*_on_ constrained to be within one standard deviation from the average *k*_on_ of wild-type concatemer. iN was constrained to be less than three times the maximal observed current of the trace being fit^15^.

### Free energies as a function of the number of I470V

To model the change in free energy as a function of the number of I470V mutations, we first determined the K_eq_ associated with each subunit when all subunits were wild-type isoleucine (K_eqI_) and when all subunits were mutated to I470V (K_eqV_). We then fit our data to a model in which K_eqI_ increases ~8% for each additional valine mutation.

## Additional information

**How to cite this article:** Venkataraman, G. *et al*. Quasi-specific access of the potassium channel inactivation gate. *Nat. Commun.* 5:4050 doi: 10.1038/ncomms5050 (2014).

## Figures and Tables

**Table 1:** Sequences of primers used in this study.

**Primer name**	**Sequence**
PCR fwd CShWT1 (+AvrII at C-term)	CCTAGGTGACTACTGGTGCAAAAGACG
PCR rev CShWT1 (+AvrII at C-term)	TAGTCACCTAGGAACGTCGGTCTCGATACTAACGG
PCR rev CShWT2 (+AvrII at N-term)	GGCAGCCCTAGGGCCACCAGGTGGG
PCR fwd CShWT2 (+AvrII at N-term)	CCTAGGGCTGCCGTTGCTCTGCGGGAG
PCR fwd CShWT3 (+KpnI at C-term)	GGTACCTGACTACTGGTGCAAAAGACG
PCR rev CShWT3 (+KpnI at C-term)	TAGTCAGGTACCAACGTCGGTCTCGATACTAACGG
PCR rev CShWT4 (+KpnI at N-term)	AGCAGCGGTACCGCCACCAGGTGGG
PCR fwd CShWT4 (+KpnI at N-term)	GGTACCGCTGCTGTTGCCCTGC
PCR fwd CShWT2 (+SgrAI at C-term)	CGCCGGCGATGACTACTGGTGCAAAAGACG
PCR rev CShWT2 (+SgrAI at C-term)	TAGTCATCGCCGGCGAACGTCTGTCTCGATACTAACGG
PCR fwd CShWT3 (+SgrAI at N-term)	CGCCGGCGAGCTGCTGTTGCCCTTCGGGAGC
PCR rev CShWT3 (+SgrAI at N-term)	AGCAGCTCGCCGGCGGCCACCAGGTGGG
PCR fwd I470V Shaker	GCTGACCGTCGCACTGCCG
PCR rev I470V Shaker	CGGCAGTGCGACGGTCAGC
PCR fwd ED192,193KK	GCAATTAATAAATTCAGAAAGAAAGAAGGC
PCR rev ED192,193KK	GCCTTCTTTCTTTCTGAATTTATTAATTGC

Fwd, forward; PCR, polymerase chain reaction; rev, reverse.
